# SUCCESSFUL TREATMENT OF *MOLLUSCUM CONTAGIOSUM* WITH A ZINC OXIDE CREAM CONTAINING COLLOIDAL OATMEAL EXTRACTS

**DOI:** 10.4103/0019-5154.70679

**Published:** 2010

**Authors:** Gilles Safa, Laure Darrieux

**Affiliations:** *From the Department of Dermatology, Centre Hospitalier de Saint-Brieuc, 22000 Saint-Brieuc, France. E-mail: gilles.safa@ch-stbrieuc.fr*

Sir,

*Molluscum contagiosum* (MC) is among the most common viral skin infections seen in children. Although the lesions may resolve spontaneously over a period of a few months or years, there are several reasons to treat them: i) the lesions can be cosmetically unappealing, ii) patients may develop a pruritic eczematous eruption, and iii) the lesions can be numerous and recurrent, especially in patients with atopic dermatitis. No therapy is universally effective. We report herein six cases of children having MC who were treated with a zinc oxide cream containing colloidal oatmeal extracts (*Avena rhealba*) with dramatic results.

Six healthy children with ≥ 10 cutaneous lesions of MC were treated in this open study. No other therapeutic procedures were undertaken with this treatment. Parents were instructed to apply the cream once daily at bed time to all the lesions for four weeks and the patients were evaluated at baseline and after four weeks.

The children were 5-11 years old (mean age: 8.1 years) and 2/6 children (33%) had a history of atopic dermatitis. At the end of the four week treatment period, 4/6 children (66%) had complete resolution of their lesions [Figures 1a and 1b] and the remainder (2 out of 6) had partial response (≥ 50% decrease in the total number of MC lesions)

**Figure 1a:**
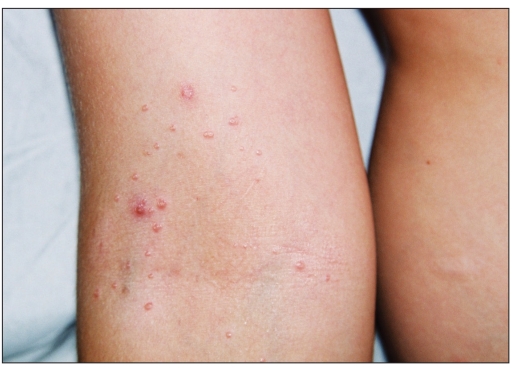
Photographs of MC before treatment

**Figure 1b:**
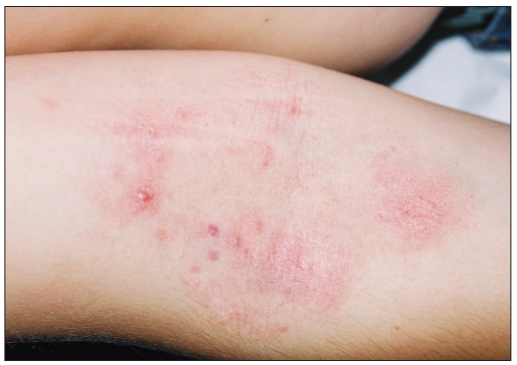
Photographs of MC after a four week treatment with a zinc oxide cream containing colloidal oatmeal extracts

MC is caused by MC virus, a DNA virus of the poxvirus family that replicates only in human epidermal keratinocytes.[1] A variety of therapies exist for the treatment of MC, and all have their own drawbacks, thus warranting the need for a non-destructive, self-administered topical therapy that is both safe and efficacious.

Taking this into consideration, we have undertaken an open trial with a zinc oxide cream containing colloidal oatmeal extracts. Colloidal oatmeal is present in a variety of dermatological and antiirritant products and has antiinflammatory properties. Recently, a study demonstrated inhibitory effects of oat extract on eicosanoid formation, expression of cytosolic phospholipase A2 (PLA2), and arachidonic acid mobilization in human keratinocytes.[2] We hypothesize that these antiinflammatory properties may play a role in the dramatic antiviral response observed in the six children in this study. Indeed, PLA2 activity was found to be critical for infectivity of some viruses such as parvoviruses.[3] In addition, studies suggested that poxviruses have an obligate requirement for arachidonic acid metabolites during replication and possess multiple genes that regulate arachidonate metabolism.[4] Obviously, randomized controlled studies are needed to confirm the effectiveness of this treatment, but its excellent safety profile appears to offer an attractive therapeutic option.

In conclusion, our findings may provide a promising new therapeutic approach to the treatment of MC and are also in agreement with recent evidence in the literature which suggests the need for therapeutic options other than curettage in patients with numerous lesions.[5]
